# miR-17-5p targets the p300/CBP-associated factor and modulates androgen receptor transcriptional activity in cultured prostate cancer cells

**DOI:** 10.1186/1471-2407-12-492

**Published:** 2012-10-24

**Authors:** Ai-Yu Gong, Alex N Eischeid, Jing Xiao, Jian Zhao, Dongqing Chen, Zhao-Yi Wang, Charles YF Young, Xian-Ming Chen

**Affiliations:** 1 Department of Medical Microbiology and Immunology, Creighton University School of Medicine, Omaha, NE, 68178, USA; 2 Key Laboratory of Biological Resource and Ecological Environment of Chinese Education Ministry, College of Life Science, Sichuan University, Chengdu, 610064, China; 3 Department of Urology, Mayo Clinic College of Medicine, Rochester, MN, 55905, USA

## Abstract

**Background:**

Androgen receptor (AR) signalling is critical to the initiation and progression of prostate cancer (PCa). Transcriptional activity of AR involves chromatin recruitment of co-activators, including the p300/CBP-associated factor (PCAF). Distinct miRNA expression profiles have been identified in PCa cells during the development and progression of the disease. Whether miRNAs regulate PCAF expression in PCa cells to regulate AR transcriptional activity is still unclear.

**Methods:**

Expression of PCAF was investigated in several PCa cell lines by qRT-PCR, Western blot, and immunocytochemistry. The effects of PCAF expression on AR-regulated transcriptional activity and cell growth in PCa cells were determined by chromatin immunoprecipitation, reporter gene construct analysis, and MTS assay. Targeting of PCAF by miR-17-5p was evaluated using the luciferase reporter assay.

**Results:**

PCAF was upregulated in several PCa cell lines. Upregulation of PCAF promoted AR transcriptional activation and cell growth in cultured PCa cells. Expression of PCAF in PCa cells was associated with the downregulation of miR-17-5p. Targeting of the 3’-untranslated region of PCAF mRNA by miR-17-5p caused translational suppression and RNA degradation, and, consequently, modulation of AR transcriptional activity in PCa cells.

**Conclusions:**

PCAF is upregulated in cultured PCa cells, and upregulation of PCAF is associated with the downregulation of miR-17-5p. Targeting of PCAF by miR-17-5p modulates AR transcriptional activity and cell growth in cultured PCa cells.

## Background

Prostate cancer (PCa) represents one of the most frequently diagnosed malignancies in men worldwide [1]. The androgen receptor (AR) is a member of the nuclear receptor superfamily that regulates ligand-dependent gene transcription [2]. Upon androgen binding, AR translocates to the nucleus and binds to consensus sequences of androgen response elements (AREs) in the genome to activate genes, such as prostate-specific antigen (PSA) [2,3]. Many of these AR-regulated genes are key regulators of prostate development and maintenance. AR signalling is also critical to the initiation and progression of PCa, and androgen-deprivation therapy remains the most prevalent treatment [2-4].

Growing evidence has shown that co-regulators, factors recruited by transcription factors to activate or repress transcription, are indispensable components of transcriptional gene regulation [5]. Under physiological conditions, co-activators are necessary for the formation of a productive transcriptional AR complex by facilitating DNA occupancy, chromatin remodeling, and/or AR protein stability and acetylation [4]. Amplification or overexpression of AR and its co-activators can sensitize cells toward a low level of androgen and has been postulated to account for aberrant AR activation in PCa [4]. In the progression of PCa, a subset of co-repressors is downexpressed [4]. Therefore, aberrant expression of co-regulators for AR may contribute to promiscuous activation of AR signaling in PCa cells. The p300/CBP-associated factor (PCAF) has been shown to act as a co-activator to regulate gene transcription, potentially including AR-regulated transcriptional activity in PCa cells [6,7]. PCAF possesses histone acetyltransferase (HAT) activity, by which it renders the chromatin environment more easily accessible for the transcriptional machinery. Apart from the acetylation of histones, HATs have been shown to acetylate AR, promoting AR transcriptional activity [5]. Nevertheless, expression of PCAF in PCa cells and its potential significance in PCa disease progression has not been fully elucidated.

MicroRNAs (miRNAs) are small, non-coding RNAs that regulate posttranscriptional gene expression based on the complementarity between miRNAs and target mRNAs. This causes either mRNA cleavage and/or translational suppression, resulting in gene suppression [8]. To date, more than 1,000 human miRNAs have been identified and, as predicted, control the expression of approximately 60% of human genes [9]. miRNAs are differentially expressed in normal and tumor cells, as well as between tumor subtypes [10,11]. Pathologically, miRNAs can be involved in the deregulation of the expression of important genes that play key roles in tumorigenesis, tumor development, and angiogenesis and have oncogenic or tumor suppressor roles [10,11]. The potential for use of miRNAs as biomarkers and therapeutic targets against cancer has been extensively studied [12,13]. Such approaches to manipulate the expression of miRNA targets in the context of disease are currently being explored in clinical trials [12]. Clinically, miRNA expression becomes altered with the development and progression of PCa [14]. Some of these miRNAs have been demonstrated to regulate the expression of cancer-related genes in PCa cells [15]. Ectopic expression of these miRNAs significantly reduced PCa growth, suggesting growth modulatory roles for these miRNAs in PCa cells [16]. A recent report demonstrates that miR-17 may target PCAF in HeLa cells [17]. Interestingly, several miRNA arrays done by different laboratories revealed an aberrant expression of miR-17 in PCa cells [18-20]. The pathogenic significance of aberrant expression of miR-17 in PCa cells is still unclear.

In this study, we investigated the expression of PCAF in PCa cells, its targeting by miR-17-5p, and its potential effects on AR transcriptional activity. The data demonstrate that PCAF is a target for miR-17-5p in PCa cells. Downregulation of miR-17-5p causes overexpression of PCAF in human PCa cells, promoting AR transcriptional activity and PCa cell growth.

## Methods

### Cell lines and reagents

RWPE1 (non-malignant prostate epithelial cells) and LNCaP, C4-2B, and PC3 PCa cells were cultured and maintained as previously reported [17-20]. PrEC (normal human prostate epithelial cells) were obtained from ATCC. The pCX-PCAF (Flag-tagged) was a gift from Dr. Tony Kouzarides (University of Cambridge, UK). PCAF siRNA was from Santa Cruz. 4, 5 α-dihydrotestosterone (DHT) (Sigma-Aldrich) was used at 10 nM, as indicated in each experiment. DHT was dissolved in ethanol, which was also used as the control vehicle.

### Western blot

Whole cell lysates were obtained from cells with M-PER Mammalian Protein Extraction Reagent (Thermo Scientific) plus several protease inhibitors (1 mM PMSF; 10 μg/mL leupeptin, 2 μg/mL pepstatin). Cell lysates were then loaded at each line (a total of 40 μg lysate proteins) in 4-12% SDS page gel to separate proteins and transferred to nitrocellulose membrane. Antibodies to PCAF (SC13124, Santa Cruz, at a final concentration of 1 μg/ml) and β-actin (A2668, Sigma-Aldrich) were used. Densitometric levels of PCAF signals were quantified and expressed as their ratio to β-actin.

### Immunocytochemistry

For immunocytochemistry staining, cells were fixed with 2% paraformaldehyde and incubated with a monoclonal antibody against PCAF (Santa Cruz, at a final concentration of 4 μg/ml), followed by anti-rabbit FITC-conjugated secondary antibody (Invitrogen) and co-staining with 4’, 6-diamidino-2-phenylindole (DAPI, 1.5 μg/ml) to stain cell nuclei. Labelled cells were assessed by fluorescence microscopy.

### Quantitative real-time PCR (qRT-PCR)

Comparative qRT-PCR was performed using the SYBR Green PCR Master Mix (Applied Biosystems). The PCR primers as follows: PCAF, forward (5^′^-CTGGAGGCACCATCTCAACGAA-3^′^) and reverse (5^′^-ACAGTGAAGACCGAGCGAAGCA-3^′^); PSA, forward (5^′^-ACCAGAGGAGTTCTTGACCCCAAA-3^′^) and reverse (5^′^-CCCCAGAATCACCCGAGCAG-3^′^); and GAPDH, forward (5^′^-TGCACCACCAACTGCTTAGC-3^′^) and reverse (5^′^-GGCATGGACTGTGGTCATGAG-3^′^). Total RNA was isolated from cells with Trizol reagent (Ambion) and treated with DNA-free Kit (Ambion) to remove any remaining DNA. qRT-PCR was performed in triplicate on the Applied Biosystems 7500 FAST Real-time PCR System. The Ct values were analyzed using the comparative Ct (ΔΔCt) method, and the target amount was obtained by normalizing to the endogenous reference (GAPDH) and relative to the control (untreated cell) [21,22]. For PCR analysis of mature miR-17-5p, total RNAs were extracted using the mirVana miRNA Isolation kit (Ambion). Hsa-miR-17-5p and snRNA RNU6B PCR primer sets were obtained from Applied Biosystems. Comparative qRT-PCR was performed in triplicate using the Taqman Universal PCR Master Mix (Applied Biosystems). Mature miR-17-5p expression level was obtained by normalizing to the endogenous reference (snRNA RNU6B) and relative to the control (untreated cell) [21,22].

### Chromatin immunoprecipitation (ChIP)

ChIP analysis was performed with a commercially available ChIP Assay Kit (Upstate Biotechnologies) in accordance with the manufacturer’s instructions. In brief, 1 × 10^6^ cells cultured in 10 cm culture dishes were cultured in the presence or absence of DHT (10 nM) for 5 h. The chromatin fraction was immunoprecipitated overnight at 4°C using anti-PCAF (Santa Cruz, at a final concentration of 2 μg/ml) or anti-AR (Santa Cruz, 2 μg/ml). A non-specific IgG (Santa Cruz) was used for control. Semi-quantitative PCR was performed with 1 μl of DNA using GoTaq Colorless Master Mix (Promega). PCR products were run in 1% Agarose gel. Densitometric levels were quantified and expressed as a ratio to the input. The primers used for the ARE-I region of the PSA promoter were forward (5^′^-TCTGCCTTTGTCCCCTAGAT-3^′^) and reverse (5^′^-AACCTTCATTCCCCAGGACT-3^′^) [23]. Primers covering the following non-ARE region of the PSA promoter were used for control: forward (5^′^-CTGTGCTTGGAGTTTACCTGA-3^′^) and reverse (5^′^-GCAGAGGTTGCAGTGAGCC-3^′^) [23].

### Anti-miR-17-5p and miR-17-5p precursor

Anti-miR-17-5p (Applied Biosystems) was used to inhibit miR-17-5p function and specific miR-17-5p precursor (pre-miR-17-5p, Applied Biosystems) to increase miR-17-5p expression [24]. Anti-miRs (anti-miR miRNA inhibitors) are commercially available, chemically modified single-stranded nucleic acids designed to specifically bind to and inhibit function of endogenous miRNAs [24]. For experiments, cells were grown to 70% confluent and treated with anti-miR or precursor to miR-17-5p (0-30 nM, Ambion) using the lipofectamine 2000 reagent (Invitrogen). Non-specific anti-miR (anti-miR-Ctrl) and precursor (precursor-Ctrl) (Ambion) were used as controls.

### Luciferase reporter constructs with PCAF 3’UTR and luciferase assay

Complementary 38 bp DNA oligonucleotides containing the putative miR-17-5p target site within the 3^′^-untranslated region (3^′^UTR) of human PCAF were synthesized with flanking SpeI and HindIII restriction enzyme digestion sites (Sense, 5^′^-CTAGGACTTGTAAATGTAATAATTAGCACTTTTGAAAA-3^′^; antisense, 5^′^-AGCTTTTTCAAAAGTGCTAATTATTACATTTACAAGTC-3^′^) and cloned into the multiple cloning site of the pMIR-REPORT Luciferase vector (Ambion). pMIR-REPORT Luciferase constructs containing mutant 3’UTR (ACTTT to AGAAT) were also generated. We then transfected cultured cells with each reporter construct (250 ng/well in a 24-well plant) and the internal pMIR-REPORT β-gal control construct (Ambion, 250 ng/well), as well as anti-miR-17-5p or precursor to miR-17-5p using the Lipofectamine 2000 reagent (Invitrogen). Luciferase activity was measured and normalized to the control β-gal level as previously reported [21,22].

### PSA luciferase reporter assay

Transfections were performed using the Lipofectamine 2000 reagent (Invitrogen). Briefly, cells (1x10^5^ cells/well) were seeded in 24-well plates. When cells grew to 70–80% confluence, 250 ng of the pGL3-PSA-6 kb luciferase reporter construct [25] and 100 ng of CMV-β-gal were transfected. After 6 h incubation, the medium was changed to the RPMI medium with 1% charcoal stripped fetal bovine serum (Life Technologies) overnight and then exposed to DHT for 24 h. Luciferase activity was measured and normalized with the β-gal.

### Cell growth

The 3-(4,5-dimethylthiazol-2-yl)-5-(3-carboxymethoxyphenyl)-2-(4-sulfophenyl)-2 H tetrazolium (MTS) assay was used to detect cell growth. The nonradioactive cell proliferation MTS Assay Kit was from Promega (Promega, WI). Cells were cultured in 24-well plates at a density of 5x10^4^ cells per well for 72 h. For measurement, 25 μL of MTS reagent was added to the medium and cells were incubated at 37°C for 1 h. The absorbance was read at 490 nm in 200 μL of soluble formazan medium with a microplate spectrophotometer. Cell number was then calculated from a standard cure and expressed as percentage of the control.

### Statistical analysis

Values are given as mean ± SE. Significance was examined by unpaired Student’s *t*-test. p < 0.05 was considered statistically significant.

## Results

### PCAF is upregulated in human PCa cell lines

**Figure 1:**
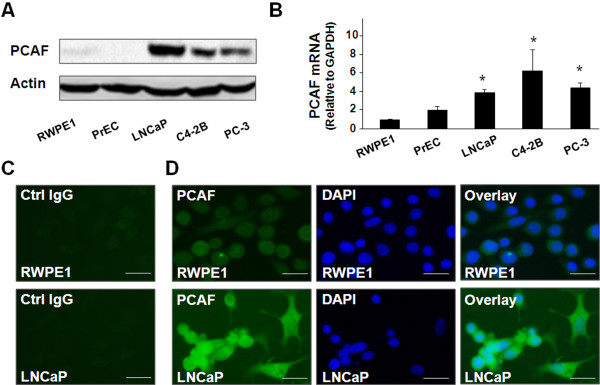
**Expression of PCAF in cultured cell lines. A** and **B**, expression of PCAF as assessed by Western blot and qRT-PCR, respectively. Representative Western blots are shown in A; β-actin was also blotted to ensure equal loading. The amount of PCAF mRNA is shown in B. Data in B are averages of three independent experiments. *, p < 0.05 compared to RWPE1 and PrEC cells. **C** and **D**, expression of PCAF protein in RWPE1 and LNCaP cells as assessed by immunofluorescent staining. PCAF was stained in green using a specific anti-PCAF antibody (in D). Cell nuclei were stained in blue with DAPI and overlaid images were also shown. The non-specific IgG was used as the control (as shown in C). Bar = 10 μm.

We first measured the PCAF expression levels by Western blot and qRT-PCR in several human PCa cell lines. As shown in Figure 1A and 1B, a significant increase of PCAF protein content was detected in these PCa cell lines, compared with two human prostate epithelial cell lines (i.e., RWPE1 and PrEC). A significant increase in PCAF mRNA levels was also found in PCa cells as assessed by qRT-PCR (Figure 1B). Increased expression of PCAF in LNCaP cells was further confirmed by immunofluorescent staining using an antibody to PCAF (Figure 1C and 1D).

### PCAF acts as a co-activator to AR and promotes DHT-stimulated AR transcriptional activity and cell growth in LNCaP cells

**Figure 2:**
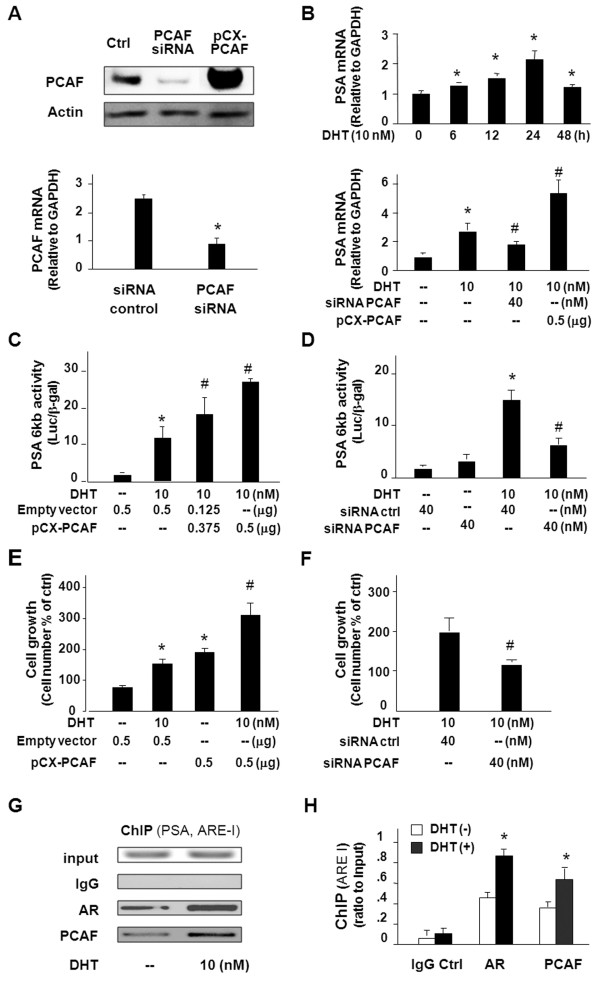
**PCAF is a co-activator to AR and promotes DHT-induced AR transcriptional activity and cell growth. A**, knockdown of PCAF by siRNA and forced expression of PCAF through transfection of the pCX-PCAF in LNCaP cells, as confirmed by Western blot and PCR analysis. **B**, functional manipulation of PCAF on DHT-induced transcription of PSA in LNCaP cells. Cells were exposed to DHT (10 nM) for up to 48 h, followed by qRT-PCR. DHT was also used to stimulate LNCaP cells that were pre-treated with siRNA to PCAF or transfected with the pCX-PCAF for 24 h. **C** and **D**, functional manipulation of PCAF on DHT-induced PSA-6 kb luciferase activity in LNCaP cells. Cells were transfected with the PSA-6 kb luciferase reporter plasmid with PCAF siRNA or pCX-PCAF for 24 h, then exposed to DHT (10 nM) for 24 h. Luciferase activity was measured and presented as the ratio to β-gal. **E** and **F**, functional manipulation of PCAF on DHT-stimulated LNCaP cell growth. Cells were transfected with pCX-PCAF (E) or treated with PCAF siRNA (F) for 24 h and then exposed to DHT (10 nM) for 72 h, followed by MTS assay. **G** and **H**, ChIP analysis of DHT-induced promoter recruitment AR and PCAF to the ARE region of the PSA gene in LNCaP cells. Cells were exposed to DHT (10 nM) for 5 h and immunoprecipitated with antibodies to AR or PCAF, or the IgG control. Specific PCR primers covering the ARE-I region of the PSA promoter were used for the PCR analysis and data were presented as ratio to the input. Data in A to F are averages of three independent experiments. *, p < 0.05 compared to non-DHT treated cells (in B, C, D, E, F and H) or siRNA control (in A). ^#^, p < 0.05 compared to cells treated with DHT only. Luc = luciferase activity.

To investigate the impact of PCAF overexpression on AR signalling in PCa cells, we used AR-responsive LNCaP cells to test the effects of functional manipulation of PCAF on DHT-induced AR transcriptional activity and cell growth. Forced expression of PCAF was carried out through transfection of cells with the pCX-PCAF construct, and a siRNA to PCAF was used to knock down PCAF expression, as shown in Figure 2A. Treatment of LNCaP cells with the PCAF siRNA attenuated DHT-induced expression of PSA as assessed by qRT-PCR (Figure 2B). In contrast, transfection of LNCaP cells with the pCX-PCAF caused a two-fold increase in DHT-induced expression of PSA gene (Figure 2B). In addition, we took a well-documented PSA 6 kb-promoter luciferase reporter assay [25,26] and tested the impact of PCAF on DHT-induced AR activity. Consistent with data from previous studies [27], DHT increased AR-associated PSA luciferase activation in LNCaP cells (Figure 2C and 2D). Transfection of cells with the pCX-PCAF enhanced DHT-induced PSA luciferase activity (Figure 2C). Complementarily, knockdown of PCAF by the PCAF siRNA partially abolished DHT-induced PSA luciferase activity (Figure 2D). Cells transfected with the empty vector or treated with the non-specific scrambled siRNA showed no changes in DHT-induced PSA luciferase activity, and pCX-PCAF and PCAF siRNA displayed a similar effect on DHT-induced PSA expression in C4-2B cells as in LNCaP cells (data not shown). Consistent with results from previous studies [27], DHT stimulated LNCaP cell growth (Figure 2E). Transfection of cells with the pCX-PCAF construct promoted DHT-induced cell growth (Figure 2E). In contrast, knockdown of PCAF through siRNA significantly decreased DHT-induced cell growth (Figure 2F).

To explore the possible mechanisms underlying PCAF-mediated AR transcriptional activation, we tested the promoter recruitment of PCAF to the AR-regulated PSA gene following DHT stimulation by ChIP analysis. AREs were previously identified in the promoter region of the PSA gene [23] and, accordingly, we used two sets of primers for the ChIP analysis: one set of primers covers the corresponding ARE-I in the promoter of the PSA gene and the other set of primers covers the non-ARE region as the control [23]. Increased promoter recruitment of AR and PCAF to the ARE region in the PSA promoter was detected in LNCaP cells following DHT stimulation (Figure 2G and 2H), whereas no binding of AR and PCAF was detected in the non-ARE region (data not shown).

### PCAF is a target for miR-17-5p in cultured PCa cells

**Figure 3:**
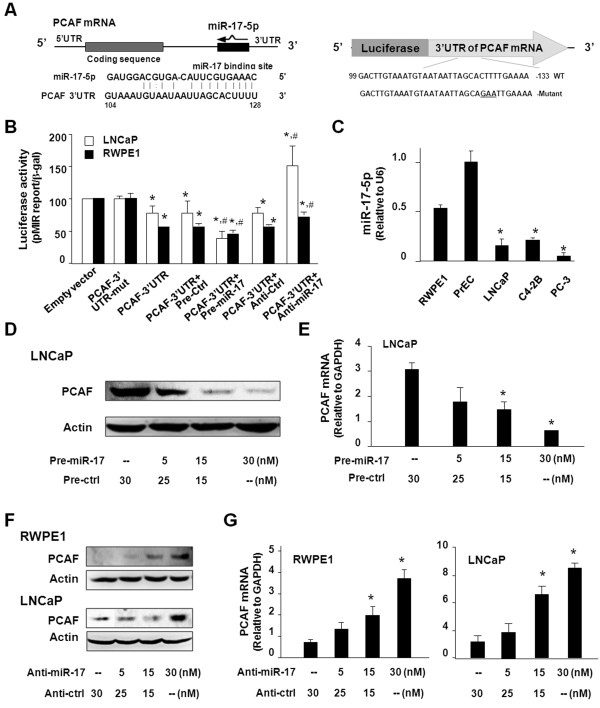
**miR-17-5p targets PCAF 3’UTR, resulting in translational suppression and RNA degradation. A**, the schematic of human PCAF mRNA showed a potential binding site in its 3’UTR for miR-17-5p. The complementary miR-17-5p-binding site in the PCAF 3’UTR was inserted to the downstream of a luciferase reporter on the pMIR-REPORT plasmid. A control plasmid with the mutant 3’UTR sequence was also generated. WT = wild-type. **B**, binding of miR-17-5p to the potential binding site in the PCAF 3’UTR results in translational suppression, as assessed by luciferase reporter assay. LNCaP and RWPE1 cells were transfected with the plasmids and treated with the anti-miR or precursor to miR-17-5p, or non-specific oligo control, for 24 h, followed by luciferase analysis. Mut = mutant; *, p < 0.05 compared to the controls; #, p < 0.05 compared to PCAF 3’UTR transfected alone. **C**, expression of miR-17-5p in cells, as assessed by qRT-CR. Mature miR-17-5p level was obtained by normalizing to the endogenous reference RNU6B. **D** and **E**, miR-17-5p precursor decreases PCAF expression in LNCaP cells. Cells were treated with various doses of miR-17-5p precursor or nonspecific precursor control, followed by Western blot for PCAF protein (after incubation for 48 h) or PCR for PCAF mRNA (after incubation for 24 h). **F** and **G**, anti-miR-17-5p increases PCAF expression in RWPE1 and LNCaP cells. Cells were treated with various doses of anti-miR-17-5p or non-specific anti-miR control followed by Western blot for PCAF protein (48 h) or PCR for PCAF mRNA (24 h). Data in B, C, E, and G are averages of three independent experiments. *, p < 0.05 compared to RWPE1 and PrEC cells (in C) or the controls in E and G.

Significant complementarity between PCAF 3’UTR and miR-17-5p has been identified in previous studies [17]. To correlate expression of miR-17-5p to PCAF upregulation in PCa cells, we generated a luciferase construct that contains the potential binding sequence of PCAF 3’UTR to miR-17-5p (Figure 3A). RWPE1 and LNCaP cells were then transfected with this construct, and luciferase activity was measured 24 h after transfection. Luciferase activity was significantly decreased in cells transfected with the PCAF 3’UTR construct with the potential binding site, compared with cells transfected with the empty control vector or the mutant 3’UTR mutant (ACTTT to AGAAT at the putative binding site), suggesting endogenous translational repression of the construct with the PCAF 3’UTR. A stronger inhibitory ratio of luciferase activity was observed in RWPE1 cells than LNCaP cells. In addition, anti-miR-17-5p markedly increased PCAF 3’UTR-associated luciferase reporter translation. In contrast, miR-17-5p precursor significantly decreased luciferase reporter translation (Figure 3B). Interestingly, a lower level of mature miR-17-5p content was detected in multiple PCa cell lines, compared with the RWPE1 and PrEC cells (Figure 3C). To test whether miR-17-5p-mediated suppression of PCAF is directly relevant to PCAF expression in prostate epithelial cells, we treated cells with miR-17-5p precursor or anti-miR-17-5p and then measured PCAF protein level (treated for 48 h) by Western blot or mRNA level (treated for 24 h) by qRT-PCR. Transfection of LNCaP cells with the miR-17-5p precursor caused a significant decrease in PCAF protein content (Figure 3D) and PCAF mRNA level (Figure 3E). Conversely, an increase in PCAF protein level and message level was detected in RWPE1 and LNCaP cells after treatment with the anti-miR-17-5p (Figure 3F and 3G).

### miR-17-5p modulates DHT-induced AR transcriptional activity and cell growth in cultured PCa cells

**Figure 4:**
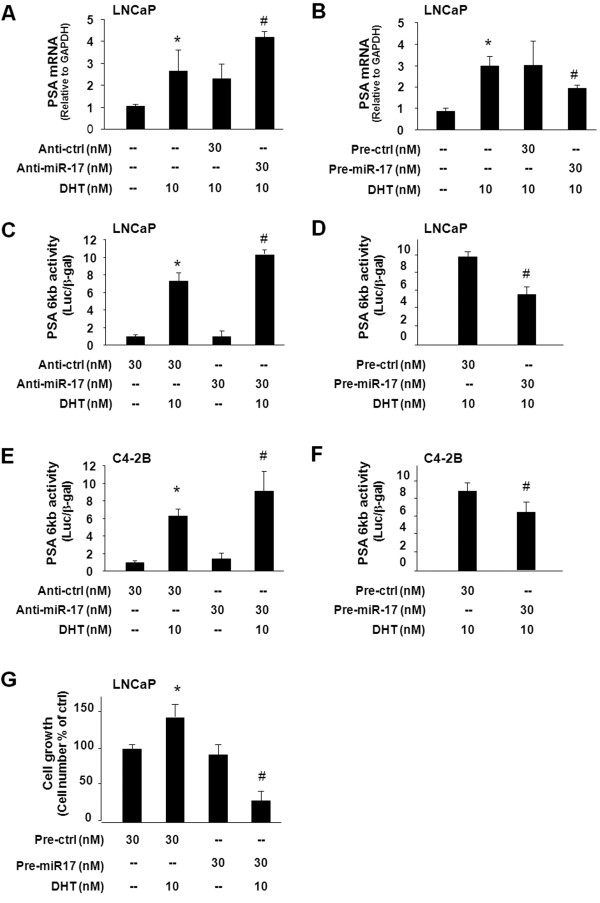
**Effects of functional manipulation of miR-17-5p on DHT-induced AR transcriptional activity and cell growth.A** and **B**, impact of functional manipulation of miR-17-5p on DHT-induced transcription of the PSA gene. LNCaP cells were treated with anti-miR-17-5p or miR-17-5p precursor for 48 h and then exposed to DHT (10 nM) for an additional 24 h, followed by qRT-PCR analysis. **C** - **F**, impact of functional manipulation of miR-17-5p on DHT-induced PSA-6 kb luciferase activity in LNCaP cells (C and D) and C4-2B cells (E and F). Cells were transfected with the PSA-6 kb luciferase reporter plasmid and simultaneously treated with anti-miR-17-5p or miR-17-5p precursor for 48 h, then exposed to DHT (10 nM) for an additional 24 h. Luciferase activity was measured and presented as the ratio to β-gal. **G**, inhibition of miR-17-5p precursor on DHT-stimulated LNCaP cell growth. Cells were treated with miR-17-5p precursor for 48 h then exposed to DHT (10 nM) for 72 h, followed by MTS assay. Data are averages of three independent experiments. *, p < 0.05 compared to non-DHT-treated cells (in A -G); ^#^, p < 0.05 compared to cells treated with DHT only. Luc = luciferase activity.

Since PCAF is a target of miR-17-5p, manipulation of miR-17-5p function should influence AR transcriptional activity and cell growth in PCa cells. Thus, we tested the effects of anti-miR-17-5p or miR-17-5p precursor on DHT-stimulated AR transcriptional activation using AR-responsive LNCaP and C4-2B cells. Treatment of LNCaP cells with anti-miR-17-5p significantly increased DHT-induced expression of PSA, as assessed by qRT-PCR (Figure 4A). In contrast, miR-17-5p precursor attenuated DHT-induced expression of the PSA gene (Figure 4B). Complementarily, effects of anti-miR-17-5p and miR-17-5p precursor on DHT-stimulated AR activity were confirmed in LNCaP cells using the PSA 6 kb-promoter luciferase assay (Figure 4C and 4D). Similar results of anti-miR-17-5p or miR-17-5p precursor on DHT-stimulated PSA 6 kb-promoter luciferase activity were obtained in C4-2B cells (Figure 4E and 4F). Moreover, precursor to miR-17-5p inhibited DHT-induced LNCaP cell growth (Figure 4G).

## Discussion

The results of our study provide the first evidence, to our knowledge, that miR-17-5p targets PCAF in PCa cells and modulates AR-regulated transcriptional activity and PCa growth. We found that PCAF is upregulated in human PCa cells and acts as a co-activator to AR and promotes DHT-stimulated AR transcriptional activity and PCa cell growth. Importantly, PCAF is a target for miR-17-5p, and upregulation of PCAF in PCa cells is associated with the downregulation of miR-17-5p. These data suggest that aberrant expression of miR-17-5p may contribute to promiscuous activation of AR signaling in PCa cells through modulation of PCAF expression at the posttranscriptional level.

Transcriptional activity of the AR is regulated by co-regulators, and the current study demonstrates that ligand-induced AR function is enhanced by PCAF in PCa cells. We found that PCAF was upregulated, both at the protein and message levels, in several PCa cell lines. The results of our luciferase reporter assay and ChIP analysis further confirmed the involvement of PCAF in the transcriptional regulation of AR-regulated PSA in cultured PCa cells, including LNCaP cells and C4-2B cells. Consequently, functional manipulation of PCAF altered ligand-induced PCa cell growth. Our data indicate that PCAF may augment AR-regulated gene expression, consistent with the results from previous studies [28,29]. Of note, it appears that both androgen-sensitive (LNCaP) and androgen-refractory (C4-2B and PC-3) cell lines showed an increase in PCAF expression. Therefore, upregulation of PCAF may not be a critical determinant for the hormone-refractory or castration-resistant emergence of the disease. Interestingly, transfection of pCX-PCAF itself stimulated LNCaP cell growth in the absence of DHT. One possible explanation for this observation is that forced expression of pCX-PCAF may also stimulate cell growth through AR-independent mechanisms.

Whereas increased transcription of the PCAF gene may be one of the mechanisms accounting for PCAF upregulation in PCa cells, our data support that miRNA-mediated posttranscriptional suppression may be involved. In a recent report by Triboulet [17], miR-17-5p was shown to bind to PCAF 3’UTR to suppress translation, resulting in suppression of the PCAF gene at the posttranscriptional level in HeLa cells. Here, we confirmed the targeting of PCAF 3’UTR by miR-17-5p using the luciferase reporter construct with the potential binding site for miR-17-5p in non-malignant prostate epithelial cells and PCa cells. A significant decrease in the luciferase reporter translation was detected in cells transfected with miR-17-5p precursor, using the luciferase reporter construct covering the potential binding site for miR-17-5p within PCAF 3’UTR. Meanwhile, we found that LNCaP cells, after transfection of the miR-17-5p precursor, also showed a decreased level of PCAF mRNA. In contrast, treatment of RWPE1 and LNCaP cells with anti-miR-17-5p increased PCAF mRNA level. Therefore, both translational repression and induction of RNA degradation are involved in miR-17-5p-mediated posttranscriptional suppression of PCAF in PCa cells. Indeed, we detected a decreased level of miR-17-5p in PCa cell lines. Although a decrease in miR-17-5p expression can partially explain the upregulation of PCAF in PCa cells through a relief of miR-17-5p-mediated posttranscriptional suppression, the majority of previous miRNA arrays done by different laboratories on prostate tumors revealed an increase in miR-17-5p expression [18,19]. Interestingly, expression of miR-17-3p, which is from the 5’ arm of the precursor for miR-17-5p, appears to be downregulated in prostate tumor tissue [20]. Obviously, alterations in the expression profile of miR-17 cluster miRNAs in PCa cells differ *in vivo* and *in vitro*, relevant to further studies on the role of miR-17 cluster in prostate tumorigenesis. In addition, this study does not preclude the possibility that other miRNAs may target PCAF in PCa cells, particularly, those miRNAs that are downregulated in PCa cells. Moreover, the question of whether the high level of PCAF protein detected in PCa cells involves dysfunction in protein degradation pathways merits further investigation.

## Conclusions

In this study, we demonstrated that PCAF is upregulated in human PCa cell lines. PCAF acts as a co-activator for AR, and promoter recruitment of PCAF enhances transactivation of AR-regulated genes in PCa cells. Upregulation of PCAF in cultured PCa cells may be associated with downregulation of miR-17-5p, a miRNA that suppresses PCAF mRNA translation and induces its degradation. Therefore, miR-17-5p targets PCAF in cultured PCa cells and modulates AR-regulated transcriptional activity and PCa growth.

## Abbreviations

PCa: Prostate cancer; AR: Androgen receptor; miRNAs: microRNAs; PCAF: p300/CBP-associated factor; DHT: Dihydrotestosterone; AREs: Androgen response elements; PSA: Prostate-specific antigen; HAT: Histone acetyltransferase; ChIP: Chromatin immunoprecipitation; 3’UTR: 3’ untranslated region; qRT-PCR: Quantitative real-time PCR.

## Competing interests

The authors declare that they have no competing interests.

## Authors’ contributions

AYG, ANE, JX, JZ, DC, and XMC participated in the study design, data collection, and data analysis and drafted the manuscript. AYG, ZYW, CYFY and XMC participated in the data analysis and drafted and revised the manuscript. All authors read and approved the final manuscript.

## Pre-publication history

The pre-publication history for this paper can be accessed here:

http://www.biomedcentral.com/1471-2407/12/492/prepub
